# Breast cancer research gaps: a questionnaire-based study to determine overall priorities and compare the priorities of patients, the public, clinicians and scientists

**DOI:** 10.1136/bmjopen-2024-084573

**Published:** 2024-08-28

**Authors:** Rebecca Louise Wilson, George Boundouki, Richard J Jackson, Rajiv V Dave, James R Harvey, Julie Wray, Laura Ballance, Julia R Henderson, Paula Duxbury, Ibrahim Ibrahim, Vivienne Appanah, Cliona C Kirwan, on behalf of the North West Breast Research Collaborative

**Affiliations:** 1 Breast Surgery, University Hospital of North Tees, Stockton on Tees, UK; 2 Breast Surgery, Royal Hallamshire Hospital, Sheffield, UK; 3 Liverpool Clinical Trials Centre, University of Liverpool, Liverpool, UK; 4 Nightingale Breast Centre, Manchester University NHS Foundation Trust, Manchester, UK; 5 Indepedent Patient Representative, Wythenshawe Hospital, Manchester, UK; 6 Wythenshawe Hospital, Manchester, UK; 7 Linda McCartney Centre, Royal Liverpool University Hospital, Liverpool, UK; 8 Research and Innovation, Greater Manchester Mental Health NHS Foundation Trust, Manchester, UK; 9 Manchester University NHS Foundation Trust, Manchester, UK; 10 The University of Manchester, Manchester, UK

**Keywords:** qualitative research, breast surgery, breast tumours, patient participation, surveys and questionnaires

## Abstract

**Abstract:**

**Objective:**

This study aims to prioritise the themes identified from the three gap analyses performed by a combination of scientists, clinicians, patients and members of the public to determine areas in breast cancer care where research is lacking. We also aimed to compare the priorities of areas of agreed research need between patients, the public, clinicians and scientists.

**Design:**

A cross-section of patients, public, clinicians and scientists completed a prioritisation exercise to rank the identified themes where research is lacking in breast cancer care.

**Participants:**

Patients, clinicians and scientists who have experienced, managed or worked in the field of breast cancer and members of the public.

**Methods:**

The research areas identified in the Breast Cancer Campaign, Association of Breast Surgery and North West Breast Research Collaborative gap analyses were outlined as 22 themes in lay terminology. Patients, members of the public, clinicians and scientists were invited to complete the prioritisation exercise, on paper or electronically, ranking the themes from 1 to 22. Comparisons were made with arithmetic mean ranking.

**Results:**

Of the 510 prioritisation exercises completed, 179 (35%) participants were patients, 162 (32%) public, 43 (8%) scientists and 122 (24%) clinicians. The theme ranked of highest priority overall was ‘better prevention’ (arithmetic mean rank 6.4 (SE 0.23)). ‘Better prevention’ was ranked top or second by patients, public and clinicians (7 (0.39), 4.7 (0.34) and 6.8 (0.5), respectively), however, scientists ranked this as their sixth most important factor (7.7 (0.92)). The public and clinicians had good agreement with patients (r=0.84 and r=0.75, respectively), whereas scientists had moderate agreement with patients (r=0.65). Certain themes were ranked significantly differently by participant groups. Compared with clinicians, patients prioritised research into ‘alternative to mammograms’, ‘diagnostic (cancer) blood test’ and ‘rare cancers’ (OR 2.1 (95% CI 1.3 to 3.5), p=0.002, OR 2.1 (95% CI 1.3 to 3.5), p=0.004 and OR 1.7 (95% CI 1.1 to 2.8), p=0.03). Compared with scientists, patients deprioritised ‘better laboratory models’ (OR 0.4 (95% CI 0.2 to 0.8), p=0.01).

**Conclusion:**

This study demonstrates that patients, public, clinicians and scientists have different research priorities, with scientists being a particular outlier. This highlights the need to ensure the engagement of patients and public in research funding prioritisation decisions.

**STRENGTHS AND LIMITATIONS OF THIS STUDY**

- Lay members of the public and patients were involved in the study design as part of the steering committee and in trialling the prioritisation exercise.
- Large numbers of patients and members of the public participated in the study.
- Relatively high agreement between patients, public and clinicians, but not scientists, strengthening the value of this prioritisation exercise.
- There was a relatively small number of participants in the scientist group compared with the other three comparator groups.

## Introduction

 Globally, 2.3 million women are diagnosed with breast cancer per year making it the world’s most prevalent cancer.^1^ With 685 000 deaths annually and 7.8 million women alive within 5 years of a breast cancer diagnosis,^1^ along with increased survival in metastatic breast cancer,^2^ there is an ongoing need for research into breast cancer prevention as well as improving quantity and quality of life in the survivorship period.

Historically, research questions were devised by scientists, however, more recently, there has been a drive to increase clinician led, research.^3^,^5^ The importance of and need for patient and public involvement (PPI) in research is now recognised globally with new initiatives at national and international levels.^6^,^8^ A UK initiative, ‘INVOLVE’, was developed in 1996 by the National Institute for Health Research^9^ with similar work being led by the Patient-Centred Outcomes Research Institute in the USA.^10^ The Independent Cancer Patients’ Voice organisation was also formed to opportune the involvement of lay members who have direct experience of cancer and an interest in being involved in research from inception to delivery.^11^ The Global PPI network is dedicated to making research an enterprise done ‘with or by’ patients and the public as opposed to ‘about or for’ them.^6^ More recently, guidelines for diversity and inclusion and checklists to optimise the impact of PPI in research have been created to facilitate this movement also.^12^,^14^

In 2012, the Breast Cancer Campaign (BCC), a UK breast cancer charity, funded a gap analysis to determine areas in breast cancer care where research is lacking.^15^ Working groups consisting of internationally renowned scientists and healthcare professionals, through an iterative process, interrogated nine main specialty areas of breast cancer. This was followed, in 2018, by a gap analysis focusing on surgical themes, led by the UK organisation, Association of Breast Surgery (ABS) and was completed in a similar manner to the BCC analysis.^16^ Despite the acknowledgement of the value of PPI, only one patient was included in these gap analyses. In 2019, the North West Breast Research Collaborative developed the 4Ps Study; identifying Public and Patient Perspectives and Priorities in breast cancer research, to investigate where Patients and the Public felt the gaps lay, and what research themes were of value. A series of patient listening events identified themes not reported in the BCC or ABS gap analyses, including how to improve breast education and the most efficient way of disseminating information.^17^ This highlighted that a difference may exist in the priorities of the patients and the public compared with scientists and clinicians.

While other studies in public health, occupational health and mental health, for example, have sought to identify the research priorities of patients,^18^,^20^ few have compared the priorities between patients and professionals.^21^

Our aim was to prioritise all of the gaps identified from the three breast cancer-specific gap analyses, to inform funding bodies and compare the priorities of patients, members of the public, scientists and clinicians.

## Methods

### Prioritisation exercise

The areas of research gaps identified in the BCC, ABS and 4Ps study gap analyses were consolidated and classified into 22 research themes, with a brief, lay description of each theme. For example, target prevention is a more accurate prediction of who is at higher or lower risk of getting breast cancer so breast screening and prevention treatments can be focused on the most at risk (table 1). This was reviewed by patient representatives to ensure appropriate understanding (JW and VA). A trial prioritisation exercise using a Likert scale (continuous visual scale from 0 to 10) was undertaken by 20 members of the public, identifying a mean (SD) score for each theme. The participants ranked the importance of each theme individually on a scale of 1–10, however, they ranked almost all of the themes highly (highest theme scored 9.7 (1.97), lowest theme scored 8.9 (0.47)). A difference of 0.8 between the highest and lowest ranked themes highlighted that a simple scoring system was inadequate to differentiate between higher and lower priorities. Participants were, therefore, asked to rank all 22 themes together in priority order from 1 (highest priority) to 22 (lowest priority). To reduce bias associated with rater fatigue and importance being given to themes higher up the list of 22 themes, 5 different formats of the same 22 themes in different orders were created and distributed (online supplemental file 1). Participants were asked to complete a version according to the day in their date of birth (eg, version 1: day of birth 1st–8th of the month, table 1). This was reviewed and trialled by the patient representative group from North West Surgical Trials Centre and local clinicians.

**Table 1:** Version 1 of prioritisation exercise

Male □ Female □ Age □ Patient □ Public □ Scientist □ Clinician □		
Rank	Area	Description
	Target prevention	More accurate prediction of who is at higher or lower risk of getting breast cancer so breast screening and prevention treatments can be focused on the most at risk.
	Better patient understanding	Improve how we explain things to people, for example explaining that they are at higher risk, or that they have breast cancer or that the breast cancer has spread.
	Better prevention	Develop better drugs to prevent breast cancer or ways to help people make lifestyle changes to prevent breast cancer. Find better ways to predict effectiveness of preventative measures.
	Alternative to mammograms	Develop better, more comfortable, more accurate machines to improve how we diagnose and monitor breast cancer.
	Safety for new equipment	Ensure checking of new treatments, materials and equipment to make sure they are safe, cost-effective and used in the best situation.
	Treating abnormal tissue	Better understand how to treat abnormalities of the breast that increase the risk of breast cancer and reduce overtreatment.
	Patient-driven help	Help patients to have a role in improving breast cancer treatment and life living with breast cancer by helping each other and doctors to understand what it is like having breast cancer.
	Impact on friends and family	Understand the impact of a breast cancer diagnosis (or a diagnosis of increased risk of BC) on friends and family.
	How does it develop	Better understand how breast cancer develops. For example: What makes a cell grow into a cancer? Why do some cancers spread and become incurable?
	Cancer blood test	Develop blood tests to diagnose and monitor breast cancer
	Predict successful treatment	Find better ways of knowing in advance which treatments will work for which patients.
	Better life in advanced cancer	Enable advanced (incurable) breast cancer patients to live as long as possible with good quality of life.
	Screening uptake	Find out what stops women from going for screening and how we can encourage more women to have screening.
	Modernise using information Technology(IT)	Develop ways to use information technology including social media to improve breast cancer screening and cancer care.
	Equality of services	Improve the efficiency of breast cancer services, ensuring all patients have access to the same services and treatments
	Increase awareness	Increase public awareness and understanding about breast cancer, especially from a younger age and find out the best method of doing this.
	Better lab models	Build better laboratory models of breast cancer to test treatments on, before they are tested on humans.
	Reduce side effects	Reduce side effects of treatments. Help breast cancer survivors and people having treatment for breast cancer to live a more normal (physical and emotional) life.
	Better clinical trials	Design better ways of doing clinical trials. For example, find out more quickly if new treatments work and if patients think the side effects are worth the benefits.
	Help patients make decisions	Understand the impact of stress on a patient’s ability to make decisions. Develop better ways of giving information to help patients make choices about their care.
	Rarer cancers	Improve how we treat unusual breast cancers such as male breast cancer, breast cancer in the very young and old, in pregnant women and rare types of breast cancer.
	Better surgery	Improve surgery so that patients need less operations, have improved outcomes, with less side effects. Work out which patients could have less surgery or no surgery at all.

BCbreast cancer

### Participants

Participants were recruited from across the UK, by approaching patients and clinical staff in UK breast units, emailing breast cancer research groups and charities and handing out the exercise to family and friends. Potential participants were either handed a paper copy or emailed a copy of the ranking exercise with a cover letter (online supplemental file 1). Returning the completed exercise implied consent.

### Statistical analysis

Continuous data are summarised as median (IQR) while categorical data are summarised as frequencies of counts with associated percentages. Missing data were rare and analyses were performed on a complete-case basis. The primary outcome is the question ranking of each outcome between 1 and 22 with the highest ranking of 1 indicating greatest importance.

Rank survey data are summarised across participant groups by calculating the arithmetic mean ranking across all participants with the associated standard error (SE). The percentage of participants to select each question in the top five positions is also presented. Graphical summaries present the mean ranking and boxplots. Comparisons of rankings which allow comparisons between participant subgroups were assessed using ordinal logistic regression. Models were adjusted for gender, patient age and survey version to account for structural imbalances between participant groups. Participant groups are analysed with ‘patients’ as the reference against which ‘public’, ‘clinician’ and ‘scientist’ rankings were compared. Results are presented in terms of ORs with associated 95% CIs with the main interest being where a participant subgroup assesses the importance of a research theme at a different level to patients. Significance is determined at the p<0.05 level. All analyses are performed by using R (V.4).

### Patient and public involvement

Patients and members of the public were included in the design, conduct and reporting of the 4Ps Study.

## Results

Between 10 December 2019 and 31 March 2021, a total of 572 questionnaires were returned, of which 62 were completed incorrectly, and therefore, 510 were included in the final analysis. 179 (35%) participants were patients, 162 (32%) were members of the public, 43 (8%) were scientists and 122 (24%) were clinicians. The majority of participants were women (418/510 (82%)) with a median (IQR) age of 49 years (37–60) (table 2). Patients are generally older than other subgroups and are more likely to be female. The majority of scientists are male.

**Table 2:** Participant demographics

Covariate	Level	Patient (n=179)	Public (n=162)	Clinician (n=122)	Scientist (n=43)	Missing (n=4)	Total (n=510)
Gender	Female	168 (94%)	135 (83%)	90 (74%)	24 (56%)	1 (25%)	418 (82%)
	Male	10 (5.6%)	25 (16%)	32 (26%)	17 (39%)	1 (25%)	85 (17%)
	Missing	1 (0.4%)	2 (1%)	0 (0%)	2 (5%)	2 (50%)	7 (1%)
Age	Median (IQR)(min, max)	56.5 (46–68)(19, 87)	46 (30.8–58) (18, 91)	41 (32–50.2) (20, 68)	41 (27–49.5) (23, 68)		49 (37–60) (18, 91)
	Missing	19	22	30	12	0	83

### Overall rankings

**Figure 1:**
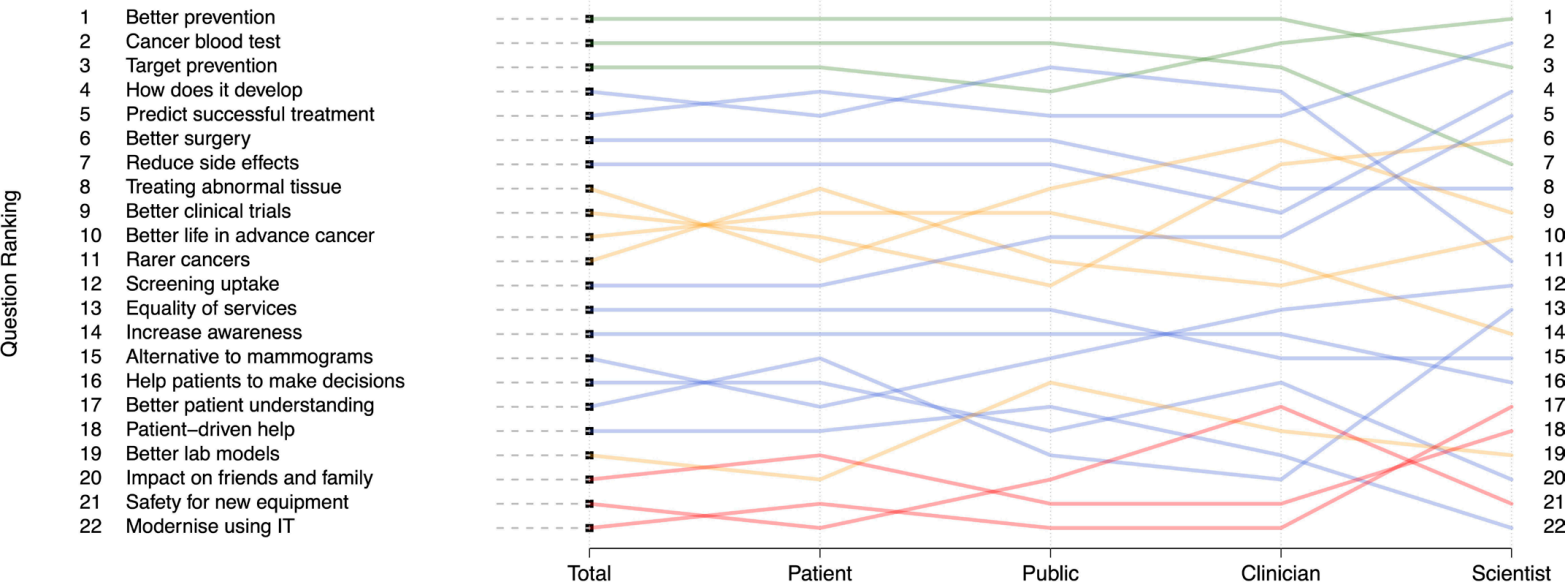
Mean rankings of each theme by participant group. The three themes with the highest importance are represented by green lines, the three themes with the lowest importance are represented by red lines. Orange lines are used to represent the five themes with the largest between subgroup heterogeneity. Blue lines represent the remaining themes.

The theme ranked top overall was ‘better prevention’ (arithmetic mean rank 6.4 (SE 0.23)), with 54% of participants including this in their top five (figure 1, online supplemental table 1). ‘Cancer blood test’ and ‘target prevention’ were the overall second and third top ranked themes, being placed in the top five by 48% and 45%, respectively. The overall three lowest ranked themes were ‘modernising use of information tehcnology (IT)’, ‘safety for new equipment’ and ‘impact on friends and family’.

### Comparison of research priorities between groups

The overall highest-ranked theme, ‘better prevention’ was ranked highest or second by patients, public and clinicians, but only sixth by scientists. Conversely, scientists’ highest research priority was ‘predict successful treatment’, which was ranked fourth, fifth and sixth by clinicians, patients and public, respectively. ‘Cancer blood test’ was ranked in the top three by patients, public and scientists, but only sixth by clinicians.

Despite ‘modernising using IT’ ‘and ‘impact on friends and family’ being specifically identified by patients and the public in the gap analysis, they were still considered low priority by all groups including patients and the public.

The themes with the largest intrasubgroup heterogeneity were ‘treating abnormal tissue’, ‘better clinical trials, ‘better life in advanced cancer’, ‘rarer cancers’ and ‘better lab models’. Perhaps unsurprisingly, an alternative to mammograms was ranked higher for patients (12th), being a top five priority for 24% (vs 18%–11% for the other groups). ‘Increase awareness’ was ranked higher (11th) by the public than either clinicians (16th) or scientists (17th).

Formal comparisons of rankings between participant groups are performed using ordinal regression techniques including gender, patient age and questionnaire version as adjusting covariates. For example, the majority of patients are female and the majority of scientists are male, therefore, any outcomes with a significant gender bias would impact any observed difference between patients and scientists. Female gender was positively associated with higher ranking of ‘reduce side effects’ and lower ranking of ‘modernise using IT’ and ‘screening uptake’. Younger age was associated with lower ranking of ‘modernise using IT’ and higher ranking of ‘better lab models’ (p=0.05) (table 3). For no theme was questionnaire version associated with ranking order and this was removed from all analyses.

**Table 3:** Comparison of priorities of public, clinicians and scientists against the patient subgroup

Theme		Gender: male vs female	Age	Public vs patient	Clinician vs patient	Scientist vs patient
Better prevention	est (SE)	0.05 (0.27)	0 (0.01)	−0.86 (0.22)	−0.11 (0.25)	−0.22 (0.39)
	OR (95% CI)	1.05 (0.62 to 1.78)	1 (0.98 to 1.02)	0.42 (0.27 to 0.65)	0.9 (0.55 to 1.46)	0.8 (0.37 to 1.72)
	P value	0.84	0.999	<0.001	0.668	0.583
Diagnostic blood test	est (SE)	0.39 (0.24)	−0.01 (0.01)	0.34 (0.21)	0.73 (0.26)	0.15 (0.34)
	OR (95% CI)	1.48 (0.92 to 2.36)	0.99 (0.97 to 1.01)	1.4 (0.93 to 2.12)	2.08 (1.25 to 3.45)	1.16 (0.6 to 2.26)
	P value	0.108	0.102	0.113	0.004	0.653
Target prevention	est (SE)	0.02 (0.25)	0 (0.01)	−0.53 (0.22)	−0.42 (0.24)	−0.18 (0.35)
	OR (95% CI)	1.02 (0.63 to 1.67)	1 (0.98 to 1.02)	0.59 (0.38 to 0.91)	0.66 (0.41 to 1.05)	0.84 (0.42 to 1.66)
	P value	0.927	0.492	0.014	0.082	0.613
How does it develop	est (SE)	−0.01 (0.26)	0 (0.01)	0.33 (0.22)	0.05 (0.25)	−0.33 (0.37)
	OR (95% CI)	0.99 (0.59 to 1.65)	1 (0.98 to 1.02)	1.39 (0.9 to 2.14)	1.05 (0.64 to 1.72)	0.72 (0.35 to 1.48)
	P value	0.981	0.426	0.132	0.842	0.364
Predict successful treatment	est (SE)	0.01 (0.25)	−0.01 (0.01)	−0.17 (0.22)	−0.35 (0.25)	−1.15 (0.37)
	OR (95% CI)	1.01 (0.62 to 1.65)	0.99 (0.97 to 1.01)	0.84 (0.55 to 1.3)	0.7 (0.43 to 1.15)	0.32 (0.15 to 0.65)
	P value	0.96	0.176	0.437	0.161	0.002
Better surgery	est (SE)	−0.14 (0.25)	0 (0.01)	−0.32 (0.21)	−0.93 (0.25)	0.33 (0.37)
	OR (95% CI)	0.87 (0.53 to 1.42)	1 (0.98 to 1.02)	0.73 (0.48 to 1.1)	0.39 (0.24 to 0.64)	1.39 (0.67 to 2.87)
	P value	0.578	0.73	0.134	<0.001	0.365
Reduce side effects	est (SE)	0.53 (0.25)	0 (0.01)	−0.36 (0.21)	−0.53 (0.25)	−1.07 (0.37)
	OR (95% CI)	1.7 (1.04 to 2.77)	1 (0.98 to 1.02)	0.7 (0.46 to 1.05)	0.59 (0.36 to 0.96)	0.34 (0.17 to 0.71)
	P value	0.034	0.486	0.096	0.032	0.004
Treating abnormal tissue	est (SE)	0.1 (0.26)	0 (0.01)	0.23 (0.21)	0.33 (0.25)	0.16 (0.37)
	OR (95% CI)	1.11 (0.66 to 1.84)	1 (0.98 to 1.02)	1.26 (0.83 to 1.9)	1.39 (0.85 to 2.27)	1.17 (0.57 to 2.42)
	P value	0.71	0.674	0.279	0.191	0.672
Better clinical trials	est (SE)	0.27 (0.25)	0.01 (0.01)	0.22 (0.22)	−0.02 (0.25)	−0.39 (0.35)
	OR (95% CI)	1.31 (0.8 to 2.14)	1.01 (0.99 to 1.03)	1.25 (0.81 to 1.92)	0.98 (0.6 to 1.6)	0.68 (0.34 to 1.34)
	P value	0.281	0.336	0.318	0.936	0.269
Better life in advanced cancer	est (SE)	0.01 (0.25)	0.01 (0.01)	0.1 (0.22)	−0.1 (0.25)	−0.14 (0.37)
	OR (95% CI)	1.01 (0.62 to 1.65)	1.01 (0.99 to 1.03)	1.11 (0.72 to 1.7)	0.9 (0.55 to 1.48)	0.87 (0.42 to 1.8)
	P value	0.983	0.119	0.659	0.696	0.7
Rarer cancers	est (SE)	0.2 (0.25)	0.01 (0.01)	0.37 (0.21)	0.54 (0.25)	1.26 (0.37)
	OR (95% CI)	1.22 (0.75 to 1.99)	1.01 (0.99 to 1.03)	1.45 (0.96 to 2.18)	1.72 (1.05 to 2.8)	3.53 (1.71 to 7.28)
	P value	0.42	0.17	0.079	0.031	0.001
Screening uptake	est (SE)	−0.57 (0.24)	0 (0.01)	−0.24 (0.21)	−0.3 (0.24)	0 (0.36)
	OR (95% CI)	0.57 (0.35 to 0.91)	1 (0.98 to 1.02)	0.79 (0.52 to 1.19)	0.74 (0.46 to 1.19)	1 (0.49 to 2.03)
	P value	0.019	0.883	0.259	0.223	0.996
Equality of services	est (SE)	0.1 (0.25)	0 (0.01)	−0.1 (0.21)	−0.17 (0.25)	−0.16 (0.38)
	OR (95% CI)	1.11 (0.68 to 1.8)	1 (0.98 to 1.02)	0.9 (0.6 to 1.37)	0.84 (0.52 to 1.38)	0.85 (0.4 to 1.79)
	P value	0.679	0.756	0.648	0.499	0.673
Increase awareness	est (SE)	−0.19 (0.25)	0 (0.01)	−0.18 (0.21)	0.44 (0.25)	0.53 (0.35)
	OR (95% CI)	0.83 (0.51 to 1.35)	1 (0.98 to 1.02)	0.84 (0.55 to 1.26)	1.55 (0.95 to 2.53)	1.7 (0.86 to 3.37)
	P value	0.453	0.811	0.404	0.073	0.125
Alternative to mammograms	est (SE)	0.33 (0.25)	−0.01 (0.01)	0.35 (0.21)	0.76 (0.25)	0.52 (0.37)
	OR (95% CI)	1.39 (0.85 to 2.27)	0.99 (0.97 to 1.01)	1.42 (0.94 to 2.14)	2.14 (1.31 to 3.49)	1.68 (0.81 to 3.47)
	P value	0.18	0.307	0.101	0.002	0.154
Help patients make decisions	est (SE)	−0.19 (0.25)	0 (0.01)	−0.2 (0.21)	−0.15 (0.25)	0.93 (0.37)
	OR (95% CI)	0.83 (0.51 to 1.35)	1 (0.98 to 1.02)	0.82 (0.54 to 1.24)	0.86 (0.53 to 1.4)	2.53 (1.23 to 5.23)
	P value	0.462	0.875	0.354	0.543	0.012
Better patient understanding	est (SE)	−0.11 (0.25)	0 (0.01)	−0.12 (0.21)	−0.14 (0.25)	0.26 (0.36)
	OR (95% CI)	0.9 (0.55 to 1.46)	1 (0.98 to 1.02)	0.89 (0.59 to 1.34)	0.87 (0.53 to 1.42)	1.3 (0.64 to 2.63)
	P value	0.648	0.471	0.586	0.563	0.463
Patient-driven help	est (SE)	0.35 (0.26)	−0.01 (0.01)	−0.28 (0.21)	−0.26 (0.25)	0.14 (0.35)
	OR (95% CI)	1.42 (0.85 to 2.36)	0.99 (0.97 to 1.01)	0.76 (0.5 to 1.14)	0.77 (0.47 to 1.26)	1.15 (0.58 to 2.28)
	P value	0.173	0.295	0.178	0.295	0.703
Better lab models	est (SE)	−0.39 (0.25)	0.01 (0.01)	0.52 (0.21)	0.14 (0.25)	−0.96 (0.37)
	OR (95% CI)	0.68 (0.41 to 1.11)	1.01 (0.99 to 1.03)	1.68 (1.11 to 2.54)	1.15 (0.7 to 1.88)	0.38 (0.19 to 0.79)
	P value	0.116	0.049	0.014	0.565	0.01
Impact on friends and family	est (SE)	0.15 (0.25)	0 (0.01)	−0.09 (0.22)	0.32 (0.25)	0.85 (0.37)
	OR (95% CI)	1.16 (0.71 to 1.9)	1 (0.98 to 1.02)	0.91 (0.59 to 1.41)	1.38 (0.84 to 2.25)	2.34 (1.13 to 4.83)
	P value	0.561	0.743	0.675	0.207	0.021
Safety for new equipment	est (SE)	−0.26 (0.25)	0 (0.01)	0.5 (0.21)	0.21 (0.25)	0.03 (0.38)
	OR (95% CI)	0.77 (0.47 to 1.26)	1 (0.98 to 1.02)	1.65 (1.09 to 2.49)	1.23 (0.76 to 2.01)	1.03 (0.49 to 2.17)
	P value	0.311	0.552	0.02	0.398	0.935
Modernise using IT	est (SE)	−0.72 (0.25)	−0.02 (0.01)	0.28 (0.21)	−0.26 (0.25)	−0.48 (0.38)
	OR (95% CI)	0.49 (0.3 to 0.79)	0.98 (0.96 to 1)	1.32 (0.88 to 2)	0.77 (0.47 to 1.26)	0.62 (0.29 to 1.3)
	P value	0.004	0.007	0.198	0.291	0.205

Results are presented in terms of estimates (SE), ORs, 95% CIs and p values. Regression analyses were adjusted for gender and age. The patient subgroup is used as a reference level. Themes where another subgroup differs significantly (p<0.05) in their ranking assessment are highlighted in green. OR>1 indicates where a subgroup gives a lower ranking than the ‘patient’ subgroup; OR<1 indicates a subgroup giving a higher ranking compared with the ‘patient’ subgroup.

Comparing priorities of public, clinicians and scientists against patient subgroups (after adjusting for gender, age and version), there was a significant difference in one theme between public and patient, five themes between clinician and patient, and six themes between scientist and patient (table 3):

- The public ranked ‘better prevention’, as of significantly greater importance than the patient group (OR −0.9 (95% CI 0.3 to 0.7), p<0.001).
- Clinicians ranked ‘better surgery’ and ‘reduce side effects’, as of significantly greater importance than the patient group (OR 0.4 (95% CI 0.2 to 0.6), p<0.001 and OR 0.6 (95% CI (0.4 to 1), p=0.032, respectively).
- Clinicians ranked ‘alternative to mammograms’, ‘diagnostic (cancer) blood test’ and ‘rare cancers’, of lower importance than the patient group (OR 2.1 (95% CI 1.3 to 3.5), p=0.002, OR 2.1 (95% CI 1.3 to 3.5), p=0.004 and OR 1.7 (95% CI 1.1 to 2.8), p=0.03, respectively).
- Scientists ranked ‘better lab models’, ‘predict successful treatment’ and ‘reduce side effects’, as of significantly higher importance than the patient group (OR 0.4 (95% CI 0.2 to 0.8), p=0.01, OR 0.3 (95% CI 0.2 to 0.7), p=0.002 and OR 0.3 (95% CI 0.2 to 0.7), p=0.004, respectively).
- Scientists ranked ‘help patients make decisions’, ‘impact on friends and family’ and ‘rare cancers’ of significantly lower importance than the patient group (OR 2.5 (95% CI 1.2 to 5.2), p=0.01, OR 2.3 (95% CI 1.1 to 4.8), p=0.02 and OR 3.5 (95% CI 1.7 to 7.3), p=0.002, respectively).

Overall, the public and clinicians have good agreement with patients (r=0.84 and r=0.75, respectively), whereas scientists have moderate agreement (r=0.65).

There was considerable intrapatient subgroup heterogeneity, with the mean ranking (and SE) demonstrating low levels of discrimination. To highlight this further, box plots for each theme are included in online supplemental file 2. These show the full distribution of rankings across subgroups. Of note is that within each group, almost all themes have a range spanning the full spectrum of rankings (1–22). This shows that there is a good degree of within-group variability in rankings applied to each theme as well as any clear differences that exist between participant groups.

## Discussion

The top three priorities identified were prevention, either improved methods of prevention or prediction of high-risk groups to focus preventative measures, and development of a blood test to facilitate early diagnosis and disease monitoring. For these themes, there was reasonably high agreement in terms of ranking between patient, public and clinicians. However, it is noteworthy that both ‘prevention’ themes were ranked lower by the scientists than the other groups. Similarly, for the lowest ranked themes, developing ways to use information technology and social media to improve screening uptake and cancer care, ensuring new techniques and devices are safe and cost-effective and understanding the impact a diagnosis has on family and friends, there was reasonable agreement between patients, public and clinicians. Scientists prioritised research into new devices much higher (16th), however, this was not a theme included in the original breast cancer care gap analysis.

The greatest variation was from the scientist subgroup, which although a smaller group still had a moderate sample size. It may be expected that scientists have different priorities from patients, given their difference in knowledge and experience of the disease and the field of their scientific research may also bias their response. As scientists are generally less patient facing, they may have more of a focus on the disease process rather than the impact a diagnosis of breast cancer has on a patient both physically and psychologically. However, some of their higher ranked themes such as ‘minimising side effects’ are themes they may have felt would benefit the patient most, despite the patients not prioritising these as highly. Equally, it is difficult to expect patients to rank highly areas of a more scientific nature which they are likely to have very little knowledge in.

Interestingly, across organisations that fund breast cancer research, the majority of the advisory board/funding panel members are scientists and predominantly male. Gender was associated with a difference in rankings of certain themes and after adjusting for the gender effect, even larger differences were detected between clinicians or scientists and patients. The male cohort was significantly smaller, 17% of participants only. It was not surprising that there were fewer male participants, especially in the patient and public groups, given that the rate of breast cancer is far higher in women than in men (1 in 8 women vs 1 in 1000 men). In retrospect, we could have tried to target men in a different way to ensure a more equal mix, especially in the public group. The ‘public’ men were likely targeted when attending the clinic with a patient, and therefore, have experience in breast cancer so may not be a true representation of male views. The results of this study can not only inform healthcare funders of priorities but also demonstrate that it is beneficial to involve patients in the funding stages of research development.

Although age is often overlooked as an element of diversity, it is well established that generational differences exist; therefore, it is unsurprising there were differences demonstrated in this study associated with age. The range of ages of the participants in this study was wide (18–91 years) allowing multigenerational experience and views. Research from Columbia University shows mixed-age teams are more effective and this is something that should also be considered in the planning and delivery of research.^22^

Despite the many reported benefits, there is still often little or no PPI in many aspects of research.^23^ This is the first study to compare research priorities between professionals and public within the field of breast cancer.

Overall, the top priorities focused on risk and prevention, followed by diagnosis and treatment. We demonstrated a good correlation between patients, public and clinicians, which was not the case in a research study comparing priorities for health research.^24^ They confirmed substantial and complex mismatches between professionals and consumer groups which were derived from distinct factors including personal values, opinions and knowledge and the values of the constituency to which the person feels they belong, differences in access to priority-setting systems and organisations and differences in terms of understandings and expectations of the research process and its results. Perhaps our more recent study represents part of a paradigm shift in healthcare to the patient-centred approach.

Manns *et al* noted differences between patients, caregivers, physicians and nurses particularly with regard to the theme symptoms when identifying research priorities in the management of kidney failure, however, the number of participants was small (n=34).^21^ Interestingly, the themes covering symptoms in our study (‘minimising side effects and quality of life in advanced cancer’) were given the highest importance by the scientist group rather than the patient group but this was not significantly different. Another small, pilot study investigating consumer priorities on evaluating health technologies compared with academics and National Health Service (NHS) staff also found major differences.^25^

Our study did not focus on why participants chose the themes that they did, however, one can hypothesise reasons behind the choices. With one in two people being diagnosed with cancer in their lifetime, it is likely the public will also have had some experience of the disease despite not suffering from it directly. Perhaps breast clinicians took a more patient-focused approach when completing the prioritisation exercise. However, despite good correlation between patients and clinicians, what clinicians perceive to be of benefit to the patient is not necessarily what the patients want as demonstrated by higher prioritisation of themes such as alternatives to mammogram and diagnostic (cancer) blood test by clinicians.

Although not a study that could be statistically powered, the large number of participants in the study increases the strength of our conclusion. Ideally, a similar proportion of scientists would have been preferred given the differences detected, but there was still a modest number in this group.

To improve the robustness of the methodology, we created five different versions of the prioritisation exercise which had the 22 themes listed in a variety of orders as we were concerned prioritising 22 themes may result in participant fatigue towards the end of the list and potentially bias results. We did not find a difference in the ranking of the themes depending on the order where the theme was placed. When comparing other common methods for research prioritisation (online crowd-voting, in-person focus groups and Delphi approach), Lavallee *et al* found the three techniques yielded similar priorities but differing perceptions of experience from the participants, with focus groups being rated the highest.^26^ Other computerised techniques could be considered for such prioritisation exercises, however, are significantly more costly.

This study has established common priorities for breast cancer research. By demonstrating differences in priorities between professionals and the public, females and males, young and old, we have confirmed it is vital to involve patients and the public and ensure diversity in all aspects of future research.

## supplementary material

10.1136/bmjopen-2024-084573 online supplemental file 1

10.1136/bmjopen-2024-084573 online supplemental file 2

## Data Availability

No data are available.
