# Supplementary material for: Breast cancer research gaps: a questionnaire-based study to determine overall priorities and compare the priorities of patients, the public, clinicians and scientists
Source: BMJ Open. 2024 Aug 28;14(8):e084573. doi: 10.1136/bmjopen-2024-084573 (PMC11367287; doi:10.1136/bmjopen-2024-084573)
Supplement: online supplemental file 1 [file bmjopen-14-8-s001.pdf]

Supplementary 1 Version 1 – 5 of the prioritization exercise

Version 1

4Ps Study

Identifying research through **P**ublic and **P**atients **P**erspectives and **P**riorities

Male ☐ Female ☐ Age: Patient ☐ Public ☐ Scientist ☐ Clinician ☐

**Rank**	**Area**	**Description**
	Target prevention	More accurate prediction of who is at higher or lower risk of getting breast cancer so breast screening and prevention treatments can be focused to the most at risk.
	Better patient understanding	Improve how we explain things to people, for example explaining that they are at higher risk, or that they have breast cancer or that the breast cancer has spread.
	Better prevention	Develop better drugs to prevent breast cancer or ways to help people make lifestyle changes to prevent breast cancer. Find better ways to predict effectiveness of preventative measures.
	Alternative to mammograms	Develop better, more comfortable, more accurate machines to improve how we diagnose and monitor breast cancer.
	Safety for new equipment	Ensure checking of new treatments, materials and equipment to make sure they are safe, cost-effective and used in the best situation.
	Treating abnormal tissue	Better understand how to treat abnormalities of the breast that increase the risk of breast cancer and reduce overtreatment.
	Patient-driven help	Help patients to have a role in improving breast cancer treatment and life living with breast cancer by helping each other and doctors to understand what it is like having breast cancer.
	Impact on friends & family	Understand the impact of a breast cancer diagnosis (or a diagnosis of increased risk of BC) on friends and family.
	How does it develop	Better understand how breast cancer develops. For example: What makes a cell grow in to a cancer? Why do some cancers spread and become incurable?
	Cancer blood test	Develop blood tests to diagnose and monitor breast cancer
	Predict successful treatment	Find better ways of knowing in advance which treatments will work in which patients.
	Better life in advanced cancer	Enable advanced (incurable) breast cancer patients to live as long as possible with good quality of life.
	Screening uptake	Find out what stops women from going for screening and how we can encourage more women to have screening.
	Modernise using IT	Develop ways to use information technology including social media to improve breast cancer screening and cancer care.
	Equality of services	Improve the efficiency of breast cancer services, ensuring all patients have access to the same services and treatments
	Increase awareness	Increase public awareness and understanding about breast cancer especially from a younger age and find out the best method of doing this.
	Better lab models	Build better laboratory models of breast cancer to test treatments on, before they are tested on humans.
	Reduce side effects	Reduce side effects of treatments. Help breast cancer survivors and people having treatment for breast cancer to live a more normal (physical and emotional) life.
	Better clinical trials	Design better ways of doing clinical trials. For example, find out more quickly if new treatments work and if patients think the side effects are worth the benefits.
	Help patients make decisions	Understand the impact of stress on a patients' ability to make decisions. Develop better ways of giving information to help patients make choices about their care.
	Rarer cancers	Improve how we treat unusual breast cancers such as male breast cancer, breast cancer in the very young and old, in pregnant women and rare types of breast cancer.
	Better surgery	Improve surgery so that patients need less operations, have improved outcomes, with less side effects. Work out which patients could have less surgery or no surgery at all.

Version 2

4Ps Study

Identifying research through **P**ublic and **P**atients **P**erspectives and **P**riorities

Male ☐ Female ☐ Age: Patient ☐ Public ☐ Scientist ☐ Clinician ☐

Rank	Area	Description
	Cancer blood test	Develop blood tests to diagnose and monitor breast cancer
	Predict successful treatment	Find better ways of knowing in advance which treatments will work in which patients.
	Better life in advanced cancer	Enable advanced (incurable) breast cancer patients to live as long as possible with good quality of life.
	Screening uptake	Find out what stops women from going for screening and how we can encourage more women to have screening.
	Modernise using IT	Develop ways to use information technology including social media to improve breast cancer screening and cancer care.
	Equality of services	Improve the efficiency of breast cancer services, ensuring all patients have access to the same services and treatments
	Increase awareness	Increase public awareness and understanding about breast cancer especially from a younger age and find out the best method of doing this.
	Better lab models	Build better laboratory models of breast cancer to test treatments on, before they are tested on humans.
	Reduce side effects	Reduce side effects of treatments. Help breast cancer survivors and people having treatment for breast cancer to live a more normal (physical and emotional) life.
	Better clinical trials	Design better ways of doing clinical trials. For example, find out more quickly if new treatments work and if patients think the side effects are worth the benefits.
	Help patients make decisions	Understand the impact of stress on a patients' ability to make decisions. Develop better ways of giving information to help patients make choices about their care.
	Rarer cancers	Improve how we treat unusual breast cancers such as male breast cancer, breast cancer in the very young and old, in pregnant women and rare types of breast cancer.
	Better surgery	Improve surgery so that patients need less operations, have improved outcomes, with less side effects. Work out which patients could have less surgery or no surgery at all.
	Target prevention	More accurate prediction of who is at higher or lower risk of getting breast cancer so breast screening and prevention treatments can be focused to the most at risk.
	Better patient understanding	Improve how we explain things to people, for example explaining that they are at higher risk, or that they have breast cancer or that the breast cancer has spread.
	Better prevention	Develop better drugs to prevent breast cancer or ways to help people make lifestyle changes to prevent breast cancer. Find better ways to predict effectiveness of preventative measures.
	Alternative to mammograms	Develop better, more comfortable, more accurate machines to improve how we diagnose and monitor breast cancer.
	Safety for new equipment	Ensure checking of new treatments, materials and equipment to make sure they are safe, cost-effective and used in the best situation.
	Treating abnormal tissue	Better understand how to treat abnormalities of the breast that increase the risk of breast cancer and reduce overtreatment.
	Patient-driven help	Help patients to have a role in improving breast cancer treatment and life living with breast cancer by helping each other and doctors to understand what it is like having breast cancer.
	Impact on friends & family	Understand the impact of a breast cancer diagnosis (or a diagnosis of increased risk of BC) on friends and family.
	How does it develop	Better understand how breast cancer develops. For example: What makes a cell grow in to a cancer? Why do some cancers spread and become incurable?

Version 3

4Ps Study

Identifying research through **P**ublic and **P**atients **P**erspectives and **P**riorities

Male ☐ Female ☐ Age: Patient ☐ Public ☐ Scientist ☐ Clinician ☐

Rank	Area	Description
	Better clinical trials	Design better ways of doing clinical trials. For example, find out more quickly if new treatments work and if patients think the side effects are worth the benefits.
	Help patients make decisions	Understand the impact of stress on a patients' ability to make decisions. Develop better ways of giving information to help patients make choices about their care.
	Rarer cancers	Improve how we treat unusual breast cancers such as male breast cancer, breast cancer in the very young and old, in pregnant women and rare types of breast cancer.
	Better surgery	Improve surgery so that patients need less operations, have improved outcomes, with less side effects. Work out which patients could have less surgery or no surgery at all.
	Target prevention	More accurate prediction of who is at higher or lower risk of getting breast cancer so breast screening and prevention treatments can be focused to the most at risk.
	Better patient understanding	Improve how we explain things to people, for example explaining that they are at higher risk, or that they have breast cancer or that the breast cancer has spread.
	Better prevention	Develop better drugs to prevent breast cancer or ways to help people make lifestyle changes to prevent breast cancer. Find better ways to predict effectiveness of preventative measures.
	Alternative to mammograms	Develop better, more comfortable, more accurate machines to improve how we diagnose and monitor breast cancer.
	Safety for new equipment	Ensure checking of new treatments, materials and equipment to make sure they are safe, cost-effective and used in the best situation.
	Treating abnormal tissue	Better understand how to treat abnormalities of the breast that increase the risk of breast cancer and reduce overtreatment.
	Patient-driven help	Help patients to have a role in improving breast cancer treatment and life living with breast cancer by helping each other and doctors to understand what it is like having breast cancer.
	Impact on friends & family	Understand the impact of a breast cancer diagnosis (or a diagnosis of increased risk of BC) on friends and family.
	How does it develop	Better understand how breast cancer develops. For example: What makes a cell grow in to a cancer? Why do some cancers spread and become incurable?
	Cancer blood test	Develop blood tests to diagnose and monitor breast cancer
	Predict successful treatment	Find better ways of knowing in advance which treatments will work in which patients.
	Better life in advanced cancer	Enable advanced (incurable) breast cancer patients to live as long as possible with good quality of life.
	Screening uptake	Find out what stops women from going for screening and how we can encourage more women to have screening.
	Modernise using IT	Develop ways to use information technology including social media to improve breast cancer screening and cancer care.
	Equality of services	Improve the efficiency of breast cancer services, ensuring all patients have access to the same services and treatments
	Increase awareness	Increase public awareness and understanding about breast cancer especially from a younger age and find out the best method of doing this.
	Better lab models	Build better laboratory models of breast cancer to test treatments on, before they are tested on humans.
	Reduce side effects	Reduce side effects of treatments. Help breast cancer survivors and people having treatment for breast cancer to live a more normal (physical and emotional) life.

Version 4

4Ps Study

Identifying research through **P**ublic and **P**atients **P**erspectives and **P**riorities

Male ☐ Female ☐ Age: Patient ☐ Public ☐ Scientist ☐ Clinician ☐

Rank	Area	Description
	Impact on friends & family	Understand the impact of a breast cancer diagnosis (or a diagnosis of increased risk of BC) on friends and family.
	How does it develop	Better understand how breast cancer develops. For example: What makes a cell grow in to a cancer? Why do some cancers spread and become incurable?
	Screening uptake	Find out what stops women from going for screening and how we can encourage more women to have screening.
	Better surgery	Improve surgery so that patients need less operations, have improved outcomes, with less side effects. Work out which patients could have less surgery or no surgery at all.
	Modernise using IT	Develop ways to use information technology including social media to improve breast cancer screening and cancer care.
	Treating abnormal tissue	Better understand how to treat abnormalities of the breast that increase the risk of breast cancer and reduce overtreatment.
	Better prevention	Develop better drugs to prevent breast cancer or ways to help people make lifestyle changes to prevent breast cancer. Find better ways to predict effectiveness of preventative measures.
	Alternative to mammograms	Develop better, more comfortable, more accurate machines to improve how we diagnose and monitor breast cancer.
	Target prevention	More accurate prediction of who is at higher or lower risk of getting breast cancer so breast screening and prevention treatments can be focused to the most at risk.
	Better lab models	Build better laboratory models of breast cancer to test treatments on, before they are tested on humans.
	Patient-driven help	Help patients to have a role in improving breast cancer treatment and life living with breast cancer by helping each other and doctors to understand what it is like having breast cancer.
	Rarer cancers	Improve how we treat unusual breast cancers such as male breast cancer, breast cancer in the very young and old, in pregnant women and rare types of breast cancer.
	Safety for new equipment	Ensure checking of new treatments, materials and equipment to make sure they are safe, cost-effective and used in the best situation.
	Cancer blood test	Develop blood tests to diagnose and monitor breast cancer
	Predict successful treatment	Find better ways of knowing in advance which treatments will work in which patients.
	Better life in advanced cancer	Enable advanced (incurable) breast cancer patients to live as long as possible with good quality of life.
	Help patients make decisions	Understand the impact of stress on a patients' ability to make decisions. Develop better ways of giving information to help patients make choices about their care.
	Equality of services	Improve the efficiency of breast cancer services, ensuring all patients have access to the same services and treatments
	Increase awareness	Increase public awareness and understanding about breast cancer especially from a younger age and find out the best method of doing this.
	Better patient understanding	Improve how we explain things to people, for example explaining that they are at higher risk, or that they have breast cancer or that the breast cancer has spread.
	Reduce side effects	Reduce side effects of treatments. Help breast cancer survivors and people having treatment for breast cancer to live a more normal (physical and emotional) life.
	Better clinical trials	Design better ways of doing clinical trials. For example, find out more quickly if new treatments work and if patients think the side effects are worth the benefits.

Version 5

4Ps Study

Identifying research through **P**ublic and **P**atients **P**erspectives and **P**riorities

Male ☐ Female ☐ Age: Patient ☐ Public ☐ Scientist ☐ Clinician ☐

Rank	Area	Description
	Rarer cancers	Improve how we treat unusual breast cancers such as male breast cancer, breast cancer in the very young and old, in pregnant women and rare types of breast cancer.
	How does it develop	Better understand how breast cancer develops. For example: What makes a cell grow in to a cancer? Why do some cancers spread and become incurable?
	Better clinical trials	Design better ways of doing clinical trials. For example, find out more quickly if new treatments work and if patients think the side effects are worth the benefits.
	Equality of services	Improve the efficiency of breast cancer services, ensuring all patients have access to the same services and treatments
	Modernise using IT	Develop ways to use information technology including social media to improve breast cancer screening and cancer care.
	Cancer blood test	Develop blood tests to diagnose and monitor breast cancer
	Better life in advanced cancer	Enable advanced (incurable) breast cancer patients to live as long as possible with good quality of life.
	Target prevention	More accurate prediction of who is at higher or lower risk of getting breast cancer so breast screening and prevention treatments can be focused to the most at risk.
	Better lab models	Build better laboratory models of breast cancer to test treatments on, before they are tested on humans.
	Patient-driven help	Help patients to have a role in improving breast cancer treatment and life living with breast cancer by helping each other and doctors to understand what it is like having breast cancer.
	Treating abnormal tissue	Better understand how to treat abnormalities of the breast that increase the risk of breast cancer and reduce overtreatment.
	Better prevention	Develop better drugs to prevent breast cancer or ways to help people make lifestyle changes to prevent breast cancer. Find better ways to predict effectiveness of preventative measures.
	Alternative to mammograms	Develop better, more comfortable, more accurate machines to improve how we diagnose and monitor breast cancer.
	Better surgery	Improve surgery so that patients need less operations, have improved outcomes, with less side effects. Work out which patients could have less surgery or no surgery at all.
	Safety for new equipment	Ensure checking of new treatments, materials and equipment to make sure they are safe, cost-effective and used in the best situation.
	Help patients make decisions	Understand the impact of stress on a patients' ability to make decisions. Develop better ways of giving information to help patients make choices about their care.
	Screening uptake	Find out what stops women from going for screening and how we can encourage more women to have screening.
	Increase awareness	Increase public awareness and understanding about breast cancer especially from a younger age and find out the best method of doing this.
	Better patient understanding	Improve how we explain things to people, for example explaining that they are at higher risk, or that they have breast cancer or that the breast cancer has spread.
	Reduce side effects	Reduce side effects of treatments. Help breast cancer survivors and people having treatment for breast cancer to live a more normal (physical and emotional) life.
	Predict successful treatment	Find better ways of knowing in advance which treatments will work in which patients.
	Impact on friends & family	Understand the impact of a breast cancer diagnosis (or a diagnosis of increased risk of BC) on friends and family.

Supplementary 2 - Box of the distribution of rankings for each survey question by participant group. These show considerable variation (heterogeneity) in each question. For example, for the question with the highest average rank (Better Prevention) showed that some participants in each group were ranking this outcome at 10th or lower.

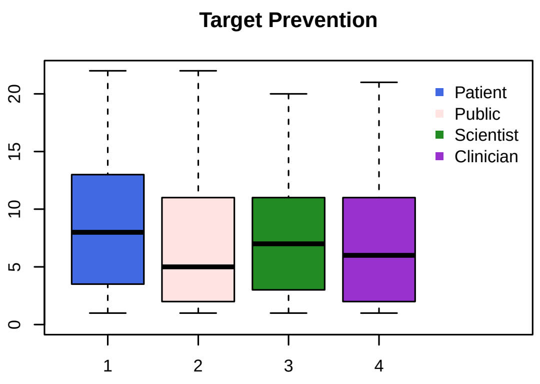
Target Prevention
Patient
Public
Scientist
Clinician
0
5
10
15
20
1
2
3
4

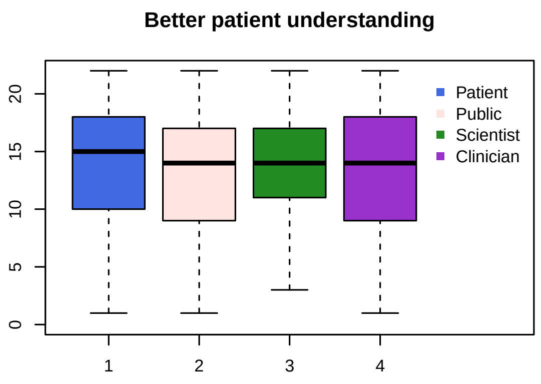
Better patient understanding
Patient
Public
Scientist
Clinician
0
5
10
15
20
1
2
3
4

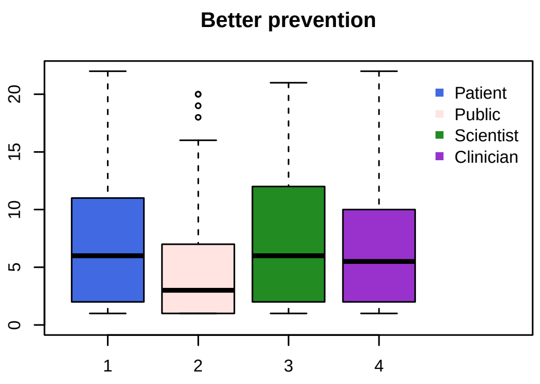
Better prevention
Patient
Public
Scientist
Clinician
0
5
10
15
20
1
2
3
4

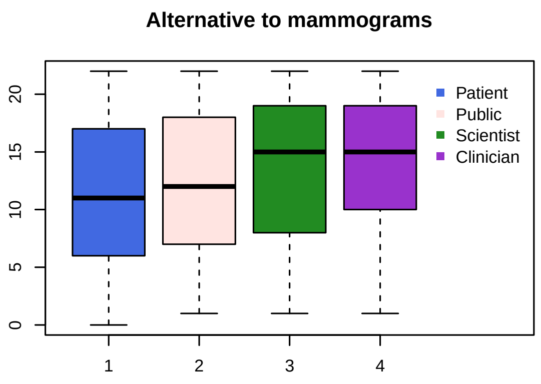
Alternative to mammograms
Patient
Public
Scientist
Clinician
0
5
10
15
20
1
2
3
4

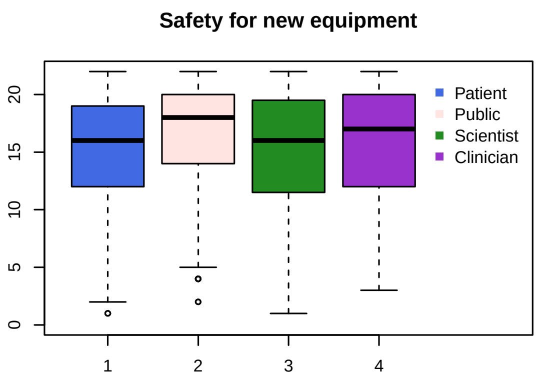
Safety for new equipment
Patient
Public
Scientist
Clinician
0
5
10
15
20
1
2
3
4

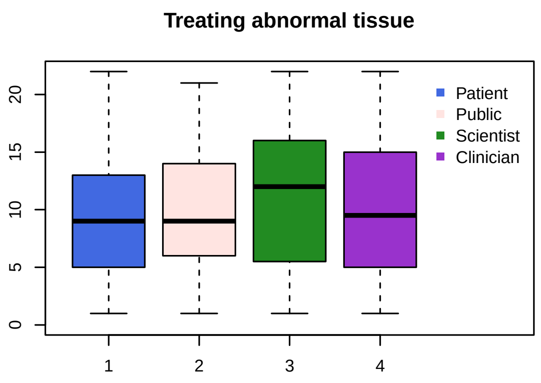
Treating abnormal tissue
Patient
Public
Scientist
Clinician
0
5
10
15
20
1
2
3
4

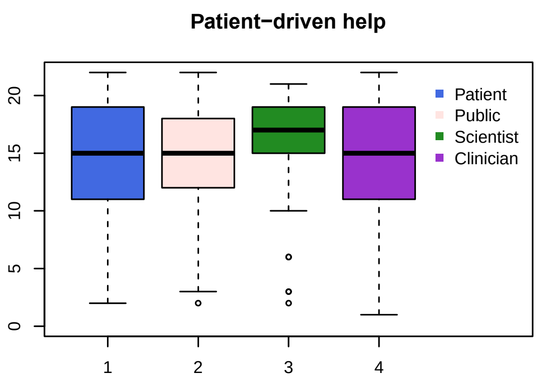
Patient−driven help
Patient
Public
Scientist
Clinician
0
5
10
15
20
1
2
3
4

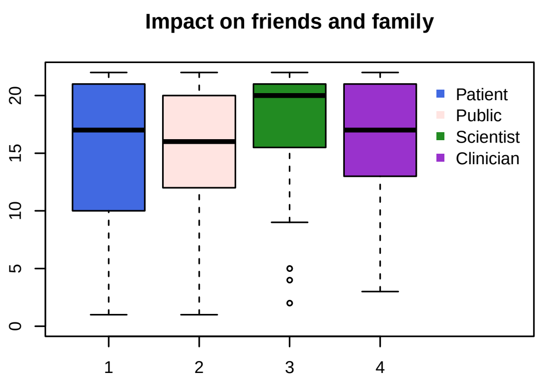
Impact on friends and family
Patient
Public
Scientist
Clinician
0
5
10
15
20
1
2
3
4

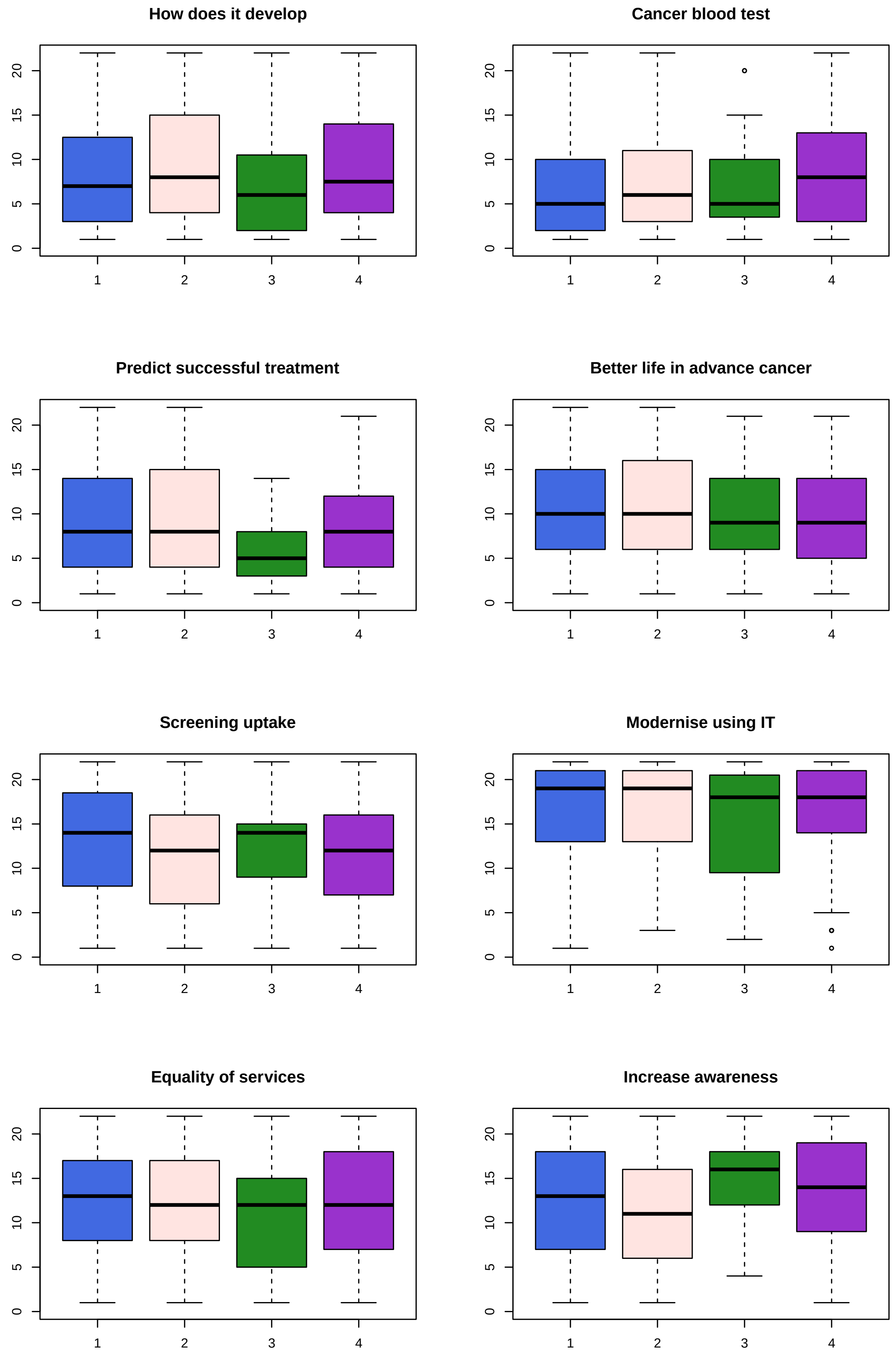
How does it develop
Cancer blood test
Predict successful treatment
Better life in advance cancer
Screening uptake
Modernise using IT
Equality of services
Increase awareness
0 5 10 15 20
1 2 3 4

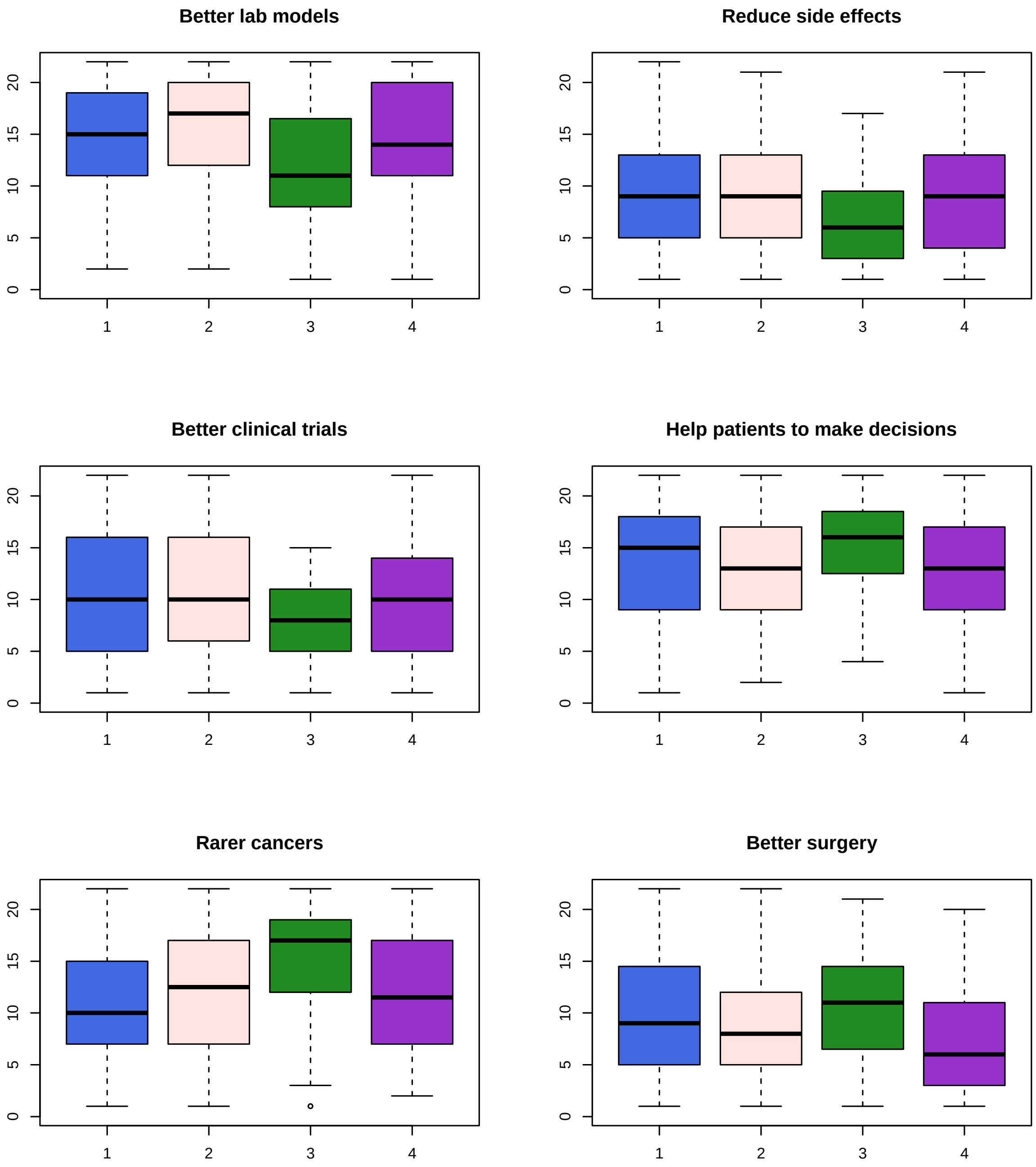
Better lab models
Reduce side effects
Better clinical trials
Help patients to make decisions
Rarer cancers
Better surgery
0
5
10
15
20
1
2
3
4
