# Supplementary material for: Breast cancer research gaps: a questionnaire-based study to determine overall priorities and compare the priorities of patients, the public, clinicians and scientists
Source: BMJ Open. 2024 Aug 28;14(8):e084573. doi: 10.1136/bmjopen-2024-084573 (PMC11367287; doi:10.1136/bmjopen-2024-084573)
Supplement: online supplemental file 2 [file bmjopen-14-8-s002.pdf]

Supplementary Table 1: Summary results by participant group. Mean (se): the arithmetic mean (standard error) of the ranking score from the combined results of each group (all participants, patients, etc) (highest importance=1; lowest = 22) Rank: Overall ranking of the question determined in each group; %select in top 5: percentage of respondents that ranked the relevant question as one of their top 5

Theme	All Participants			Patients			Public			Clinicians			Scientists		
	Mean (se)	Rank	% select in top 5	Mean (se)	Rank	% select in top 5	Mean (se)	Rank	% select in top 5	Mean (se)	Rank	% select in top 5	Mean (se)	Rank	% select in top 5
Better prevention	6.3 (0.23)	1	54	7 (0.39)	2	48	4.7 (0.34)	1	65	6.8 (0.5)	1	50	7.7 (0.92)	6	42
Diagnostic blood test	7.5 (0.26)	2	48	6.5 (0.43)	1	56	7.5 (0.44)	3	46	9 (0.59)	6	39	6.7 (0.68)	2	51
Target prevention	7.7 (0.26)	3	45	8.7 (0.45)	4	40	7.1 (0.46)	2	52	7.3 (0.49)	2	43	7.5 (0.78)	5	40
How does it develop	8.8 (0.28)	4	38	8.6 (0.46)	3	39	9.8 (0.52)	7	33	8.6 (0.54)	5	40	7.1 (0.94)	4	49
Predict successful treatment	8.9 (0.25)	5	35	9.4 (0.44)	5	34	9.4 (0.46)	6	33	8.4 (0.47)	4	32	6.1 (0.56)	1	53
Better surgery	9.1 (0.25)	6	32	10 (0.44)	8	28	8.9 (0.42)	4	30	7.4 (0.46)	3	44	11 (0.84)	10	21
Reduce side effects	9.2 (0.23)	7	29	9.7 (0.41)	7	28	9.3 (0.39)	5	27	9.3 (0.47)	7	31	7 (0.73)	3	42
Treating abnormal tissue	10.1 (0.25)	8	25	9.7 (0.4)	6	26	9.9 (0.41)	8	23	10.3 (0.54)	10	27	11.1 (0.95)	11	26
Better clinical trials	10.1 (0.26)	9	26	10.4 (0.45)	9	27	10.8 (0.47)	9	23	9.9 (0.51)	9	28	8 (0.58)	7	28
Better life in advanced cancer	10.4 (0.26)	10	25	10.6 (0.42)	10	23	10.8 (0.48)	10	23	9.7 (0.5)	8	29	10 (0.84)	8	23
Rarer cancers	11.8 (0.26)	11	17	10.7 (0.41)	11	17	12.1 (0.46)	13	19	12.1 (0.53)	13	16	14.9 (0.84)	18	9
Screening uptake	12.1 (0.27)	12	18	12.8 (0.48)	15	17	11.5 (0.47)	12	19	11.6 (0.51)	11	20	12.3 (0.85)	13	12
Equality of services	12.2 (0.27)	13	18	12.4 (0.45)	14	17	12.3 (0.45)	14	15	12 (0.59)	12	20	10.9 (0.95)	9	28
Increase awareness	12.5 (0.27)	14	17	12.1 (0.47)	13	19	11.3 (0.48)	11	22	13.6 (0.54)	16	13	14.7 (0.78)	17	7
Alternative to mammograms	12.5 (0.28)	15	18	11.3 (0.49)	12	24	12.4 (0.49)	15	18	14.1 (0.52)	17	11	13.4 (0.94)	14	14
Help patients make decisions	13.3 (0.24)	16	12	13.5 (0.4)	16	10	12.9 (0.42)	16	13	12.8 (0.52)	14	16	15.2 (0.69)	19	5
Better patient understanding	13.4 (0.23)	17	10	13.7 (0.4)	17	9	13.2 (0.41)	17	10	12.8 (0.49)	15	12	14 (0.68)	15	5
Patient driven help	14.5 (0.22)	18	7	14.5 (0.38)	19	6	14.4 (0.37)	18	7	14.3 (0.49)	18	7	15.8 (0.77)	21	7
Better lab models	14.7 (0.24)	19	6	14.5 (0.39)	18	7	15.8 (0.4)	20	4	14.6 (0.5)	19	4	11.8 (0.89)	12	16
Impact on friends & family	15.6 (0.26)	20	8	15.1 (0.48)	20	12	15.4 (0.42)	19	6	16 (0.51)	21	7	17.8 (0.79)	22	7
Safety for new equipment	15.6 (0.23)	21	6	15.1 (0.4)	21	7	16.3 (0.38)	21	5	15.8 (0.47)	20	5	14.6 (0.94)	16	12
Modernise using IT	16.6 (0.24)	22	6	16.7 (0.41)	22	7	17 (0.43)	22	7	16.6 (0.47)	22	5	15.3 (0.91)	20	5
