# Supplementary material for: Breast cancer research gaps: a questionnaire-based study to determine overall priorities and compare the priorities of patients, the public, clinicians and scientists
Source: BMJ Open. 2024 Aug 28;14(8):e084573. doi: 10.1136/bmjopen-2024-084573 (PMC11367287; doi:10.1136/bmjopen-2024-084573)
Supplement: Supplementary file 3 [file bmjopen-2024-084573.reviewer_comments.pdf]

PEER REVIEW HISTORY

BMJ Open publishes all reviews undertaken for accepted manuscripts. Reviewers are asked to complete a checklist review form and are provided with free text boxes to elaborate on their assessment. These free text comments are reproduced below.

ARTICLE DETAILS

**TITLE (PROVISIONAL)**	Breast cancer research gaps: a questionnaire-based study to determine overall priorities and compare the priorities of patients, the public, clinicians and scientists
**AUTHORS**	Wilson, Rebecca; Boundouki, George; Jackson, R; Dave, Rajiv; Harvey, James; Wray, Julie; Ballance, Laura; Henderson, Julia; Duxbury, Paula; Ibrahim, Ibrahim; Appanah, Vivienne; Kirwan, Cliona

VERSION 1 - REVIEW

**REVIEWER NAME**	Seely, Jean
**REVIEWER AFFILIATION**	University of Ottawa, Radiology
**REVIEWER CONFLICT OF INTEREST**	None
**DATE REVIEW RETURNED**	16-May-2024

**GENERAL COMMENTS**	This study in an innovative approach to the evaluation of the priorities of patients, public, clinicians and scientists with respect to breast cancer research and priorities.<br><br>The strength of including patients in assessing priorities was identified correctly as a gap to be rectified. The purpose was to compare the priorities between patients and professionals, including clinicians and scientists. Using a ranking of priority was a helpful way to distinguish between the highest and lowest priorities given that all were highly rated.<br>The results were interesting in that scientists, the smallest group studied, ranked more highly the “help patients make better decision’, ‘impact on family and friends’ and ‘rare cancers’ more highly than patients and clinicians, suggesting a disconnect between scientific knowledge about what patients and clinicians value most. In addition, patients and clinicians placed higher value on a blood test to evaluate early diagnosis and monitoring, and this should inform the direction that scientists should take in future research. It attests to the need for more patient-centred approach to drive the research direction. This has been adopted in clinical care and should be adopted more systematically in research policy.<br><br>The limitation as noted by the authors is that the scientist group was smallest, and there was more heterogeneity in the group. There were 179 participant patients, 162 members of the public, 122 clinicians and only 43 scientists. Given that the most interesting part of the study was the difference between the scientists and the patients, public and clinicians, this is a major limitation. The similarities between the patients, public and clinicians should be noted as a strength. My concern is that the smaller sample size of scientists may make the results less reliable. Nonetheless, the authors found significant differences among the groups with respect

	to the top-rated priority, indicating that the study results were sufficient.<br><br>What is interesting is that prevention ranked top 1 or 2 among patients, public, and clinicians but only 6th among scientists, while scientists ranked predicting successful treatment their top priority. Similarly, there was good agreement among public and clinicians with patients whereas scientists had only moderate agreement.<br><br>There is an error in the incidence of breast cancer, it should be 1 in 8, not 1 in 9 line 47 page 12.<br><br>The authors comment on the fact that they did not evaluate why the participants chose the themes they did but they hypothesized some reasons, e.g., the prevalence of breast cancer among the population. They also commented on why the clinicians may have been more aligned with the patients. However, what is lacking are potential reasons why the scientists responded differently to the patients, public and clinicians. The authors should provide some explanations for the scientists' responses. Is this because of a lack of "skin in the game" or some other reason? Are the scientists too removed from patients to understand the patient, clinical and public priorities? The authors allude to this by suggesting that more patients need to be involved in setting research funding priorities, however, should add this to manuscript.

**REVIEWER NAME**	Li, Min
**REVIEWER AFFILIATION**	Shanxi Bethune Hospital, Shanxi Academy of Medical Sciences, Tongji Shanxi Hospital, Third Hospital of Shanxi Medical University
**REVIEWER CONFLICT OF INTEREST**	No relevant conflicts of interest
**DATE REVIEW RETURNED**	20-May-2024

**GENERAL COMMENTS**	1. The research method is not clear and rigorous. If we can clarify the specific investigation methods and contents, the article will be more complete.<br>2. How does the research ensure a comprehensive investigation of the research fields of breast cancer involving three groups of people?<br>3. This study is of little significance for clinical guidance.

VERSION 1 – AUTHOR RESPONSE

Reviewer: 1

Thank you for your helpful and detailed comments

Re: The similarities between the patients, public and clinicians should be noted as a strength.

Thank you for suggesting this. We have now included it in the strengths and limitations of the study 'Relatively high agreement between patients, public and clinicians but not scientists, strengthening the value of this prioritisation exercise. Pg 9 lines 11-14.

We have also reflected on this in the discussion, adding to the paragraph discussing the good correlation between patients, public and clinicians 'Perhaps our more recent study represents part of a paradigm shift in health care to the patient-centred approach.' Pg 19 lines 1-3

Re: There is an error in the incidence of breast cancer, it should be 1 in 8, not 1 in 9 line 47 page 12.

We have now amended this to '1 in 8'. Pg 20, line 3

Re: However, what is lacking are potential reasons why the scientists responded differently to the patients, public and clinicians. The authors should provide some explanations for the scientists' responses. Is this because of a lack of "skin in the game" or some other reason? Are the scientists too removed from patients to understand the patient, clinical and public priorities? The authors allude to this by suggesting that more patients need to be involved in setting research funding priorities, however, should add this to manuscript.

Thank you for highlighting this. We have provided some explanations in the discussion 'It may be expected that scientists have different priorities to patients, given their difference in knowledge and experience of the disease and the field of their scientific research may also bias their response. As scientists are generally less patient facing they may have more of a focus on the disease process rather than the impact a diagnosis of breast cancer has on a patient both physically and psychologically. However, some of their higher ranked themes such as 'minimising side effects' are themes they may have felt would benefit the patient most, despite the patients not prioritising these as highly. Equally, it is difficult to expect patients to rank highly areas of a more scientific nature which they are likely to have very little knowledge in.' Pg 18 lines 33-49.

Reviewer: 2

Thank you for your comments

1. The research method is not clear and rigorous. If we can clarify the specific investigation methods and contents, the article will be more complete.

We are unsure what is not clear in the methods, but have attempted to improve it by adding an example of a theme from the prioritisation exercise, the whole contents of which is displayed in table 1'For example, target prevention - more accurate prediction of who is at higher or lower risk of getting breast cancer so breast screening and prevention treatments can be focused to the most as risk ….' pg 10 lines 58-60.

We also added further details to the trial methods 'The participants ranked the importance on each theme individually on a scale of one to ten, however they ranked almost all of the themes highly (highest theme scored 9.7 (1.97), lowest theme scored 8.9 (0.47))' and 'Participants were therefore asked to rank all 22 themes together in priority order from 1 (highest priority) to 22 (lowest priority)'. Pg 11 line 5 and 14

2. How does the research ensure a comprehensive investigation of the research fields of breast cancer involving three groups of people?

We are unclear what is meant here. We presume the ‘three groups of people’ are the three original studies that informed the identification of the themes. The Breast Cancer care (BCC) gap analysis was performed by internationally renowned scientists, clinicians and healthcare professionals. The Association of Breast Surgery gap analysis was also performed by internationally renowned expert clinicians in their field along with a patient representative from independent patient voice but focused on the surgical aspects of breast cancer care which were not covered in the BCC analysis. The 4Ps gap analysis was performed by breast cancer past or current patients and the public. Including the findings from all three groups ensures a comprehensive appreciation of the gaps in research of breast cancer care available in the literature. Further detail has been added to the introduction where these three studies are discussed to reflect this, ‘In 2012 the Breast Cancer Campaign (BCC), a UK breast cancer charity, funded a gap analysis to determine areas in breast cancer care where research is lacking15. Working groups consisting of internationally renowned scientists and healthcare professionals, through an iterative process, interrogated nine main speciality areas of breast cancer. This was followed, in 2018, by a gap analysis focusing on surgical themes, led by the UK organisation, Association of Breast Surgery (ABS) and completed in a similar manner to the BCC analysis16. Pg 9 line 60 and pg 10 lines 1-7

3. This study is of little significance for clinical guidance.

The document is not intended as clinical guidance. It is intended to provide guidance to funding bodies and researchers, highlighting the need for patient and public involvement when developing research calls, research proposals and allocating grant funding.

VERSION 2 – REVIEW

**REVIEWER NAME**	Seely, Jean
**REVIEWER AFFILIATION**	University of Ottawa, Radiology
**REVIEWER CONFLICT OF INTEREST**	None
**DATE REVIEW RETURNED**	16-Jul-2024

**GENERAL COMMENTS**	Thank you for addressing the concerns raised. This is satisfactory for publication.
